# An Extended Modular Processing Pipeline for Event-Based Vision in Automatic Visual Inspection

**DOI:** 10.3390/s21186143

**Published:** 2021-09-13

**Authors:** Moritz Beck, Georg Maier, Merle Flitter, Robin Gruna, Thomas Längle, Michael Heizmann, Jürgen Beyerer

**Affiliations:** 1 Fraunhofer IOSB, Karlsruhe, Institute of Optronics, System Technologies and Image Exploitation IOSB, 76131 Karlsruhe, Germany; bkmoritz@web.de (M.B.); merle.flitter@iosb.fraunhofer.de (M.F.); robin.gruna@iosb.fraunhofer.de (R.G.); thomas.laengle@iosb.fraunhofer.de (T.L.); juergen.beyerer@iosb.fraunhofer.de (J.B.); 2 Institute of Industrial Information Technology (IIIT), Karlsruhe Institute of Technology (KIT), 76131 Karlsruhe, Germany; michael.heizmann@kit.edu; 3 Vision and Fusion Laboratory (IES), Karlsruhe Institute of Technology (KIT), 76131 Karlsruhe, Germany

**Keywords:** event-based vision, automatic visual inspection, dynamic vision sensors, object classification

## Abstract

Dynamic Vision Sensors differ from conventional cameras in that only intensity changes of individual pixels are perceived and transmitted as an asynchronous stream instead of an entire frame. The technology promises, among other things, high temporal resolution and low latencies and data rates. While such sensors currently enjoy much scientific attention, there are only little publications on practical applications. One field of application that has hardly been considered so far, yet potentially fits well with the sensor principle due to its special properties, is automatic visual inspection. In this paper, we evaluate current state-of-the-art processing algorithms in this new application domain. We further propose an algorithmic approach for the identification of ideal time windows within an event stream for object classification. For the evaluation of our method, we acquire two novel datasets that contain typical visual inspection scenarios, i.e., the inspection of objects on a conveyor belt and during free fall. The success of our algorithmic extension for data processing is demonstrated on the basis of these new datasets by showing that classification accuracy of current algorithms is highly increased. By making our new datasets publicly available, we intend to stimulate further research on application of Dynamic Vision Sensors in machine vision applications.

## 1. Introduction

**Figure 1:**
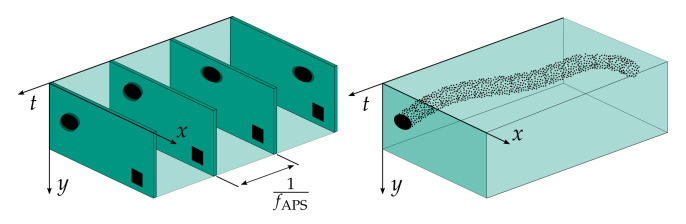
Difference between the frame (**left**) and event-based (**right**) vision technology. The scene shows a sphere moving from the right to left image border and a static square in the lower right corner. A frame-based camera perceives the square as well as the sphere with a motion blur at constant sampling times. The event-based camera does not suffer from motion blur and generates an asynchronous event stream at the edge of the sphere with high temporal resolution. However, the static square is not perceived.

In recent years, a new type of image sensor principle has undergone a rapid development. So-called Dynamic Vision Sensors (DVS) merely perceive changes in intensity and encode this information as events in an asynchronous stream. The approach is further illustrated in Figure 1. DVS promises high time resolution in the order of microseconds, low latency, and a low data rate by omitting transmission of redundant information, i.e., static regions of the image plane. If there is no moving object in the visual scope, no events are triggered. Although theoretical advantages of the sensor principle have been discussed thoroughly [1], their fields of application still remain somewhat unclear. In previous works, DVS are mainly investigated in the field of autonomous driving, for monitoring, and gesture recognition. Additionally, it has recently been shown that the strength of the concept is particularly evident in sparsely populated scenes [2]. However, this does match very well to the fields of application considered so far.

Automatic visual inspection is a widely spread form of automatic quality control. It is vital to a variety of processing and manufacturing industries and serves the purpose of inspecting wrought materials and end products, among others. In contrast to manual inspection performed by human personnel, machine vision-based approaches promise objective and reproducible results, fast inspection execution, and less expensive solutions. By “looking”, it is evaluated whether components have been correctly assembled, containers are fully loaded, or individual objects meet the quality requirements in terms of dimensions, shape, surface finish, or color [3], to name a few examples. The process of “looking” is performed by one or multiple imaging sensors. According to the state-of-the-art, those are typically either line-scanning or frame-based.

In this paper, application of DVS in the so far unconsidered field of automatic visual inspection is investigated. Machine vision applications are often characterized by a highly controlled environment. This is reflected, among other things, in a static sensor position, control over the design and parameterization of the illumination, and the feeding or presentation of test objects. This in turn enables the realization of data acquisition conditions in which the potential of DVS can fully unfold, for instance, sparsely populated scenes. A resulting advantage lies in increasing the processing speed of “looking”.

Main contributions of this paper are threefold. First, the most recent classification algorithms are evaluated in the context of the new application domain of automatic visual inspection in a unitized, modular machine vision pipeline. Second, a novel algorithmic module is proposed, included and evaluated using the pipeline in order to meet common challenges in automated visual inspection, such as arbitrary object rotations. We refer to this algorithmic contribution as *contrast-based windowing* and demonstrate how it enhances classification accuracy distinctly. Third, two novel event-based datasets for challenging scenarios in automatic visual inspection are introduced. By publishing the datasets alongside this paper, we intend to further stimulate research on DVS in the context of machine vision.

This paper is organized as follows. Following this brief introduction, related work is reviewed in Section 2. A modular pipeline for event processing in the context of machine vision is presented in Section 3. Here, the new concept of contrast-based windowing is also introduced. Following, two novel datasets containing event-streams from typical visual inspection scenarios are introduced in Section 4. Results from utilizing the presented pipeline with these datasets are presented in Section 5. Section 6 concludes the paper.

## 2. Related Work

In the context of machine vision, modalities of image acquisition, preprocessing, information compression, and decision making are of particular interest. In addition to the obviously different sensor modalities for image acquisition, it is important to note that conventional image processing methods are not applicable for DVS due to the fundamentally different data format. In the following, related work addressing the above-mentioned is reviewed in Section 2.1. Particular attention is given to existing event-based datasets and the fields of application they origin from in Section 2.2.

### 2.1. Event-Based Image Processing

A fairly up-to-date overview of currently available cameras is provided in [1]. Latest models feature up to 1280×960 pixels and distinguish themselves from early models by an increased bandwidth. Available models further differ in the specified latency, which is mainly due to the different readout mechanisms. Some models also include an integrated *Inertial Measurement Unit* (IMU), which is mainly used in mobile applications. Generally, three different variants of DVS can be distinguished: First, native DVS chip technology involves the plain event generation by binary sampling of the logarithmic intensity response [4]. Second, *Dynamic and Active Pixel Vision Sensors* (DAVIS) additionally offer the possibility of recording conventional frames at a constant frequency or asynchronously on demand [5]. Both methods are implemented on one chip, which allows a high fill factor and a compact design. A disadvantage of this method is the low dynamic range for the conventional frames [1]. Third, the circuits of *Asynchronous Time-Based Image Sensors* (ATIS) pixels are extended by a photodiode, which is used to determine the time difference between two generated events [6]. This way, an absolute intensity value is determined and featured in the event information [7]. Disadvantages of this design are the high space requirement of a pixel on the chip and erroneous intensity values in dark scenes [1].

An important task in data preprocessing is noise filtering. Raw event streams suffer from two different types of noise. The first type is background activity noise. It is mainly caused by thermal noise [1] and losses in transistor switching operations [8]. The useful signal distinguishes from this kind of noise by being strongly correlated in space and time. The second type of noise is so-called hot pixels, which generate events at a constantly high rate. In most cases, this is due to a faulty reset switch [8]. To reduce background noise, correlation filters are mainly used in a spatio-temporal plane, as, for instance, in [9]. This approach is extended by a learning algorithm that filters out high-frequency, i.e., hot pixels [9]. In the field of automatic visual inspection, it may be expected that good results can be achieved with simple, high-performance filtering methods due to the constant image acquisition conditions.

In order to assign information of individual events to an object, an event-based object detection and tracking method is needed. Proposed algorithms can be divided into three groups. The first group includes trackers that update the position asynchronously when an event occurs. Different methods have been proposed, including an Iterative Closest Point Algorithm (ICP) [10] and Mean-Shift [11] with a Gaussian kernel. The second group refers to algorithms that accumulate events over a time horizon and adjust the position based on a motion model, for instance by means of Expectation-Maximization Algorithms (EM) [12] and accumulated images [13]. The third group includes algorithms that fuse the high temporal resolution of events and the high spatial accuracy of conventional images, such as in [14]. In this work, we focus on the asynchronous processing of events, i.e., the first mentioned group, which comes closest to the basic principle of the camera and does not require temporal accumulation within a time window.

Approaches for object classification can also be clustered into three groups. One possibility is to reconstruct an intensity image based on the events and subsequently apply conventional image classification methods. In addition to integrating methods, such as Poisson reconstruction [1] and a complementary filter [15], data-driven algorithms are increasingly used for this purpose [16]. Instead of performing classification based on a reconstructed image, there are approaches where events are first transformed into a feature map. Different transformation rules have been proposed to transfer events into a two-dimensional feature space. The method of Lagorce et al. [17] considers hierarchical models of time-surfaces that are composed of the most recent timestamps of all past events in its neighborhood. The *Histogram of Averaged Time Surfaces* (HATS) method [18] uses a similar approach, yet the formation of local histograms leads to better results. With HATS, events within constant time intervals are considered. A new method by Cannici et al. [19] takes up this local consideration and extends the idea to also learn the time horizon using *Long short-term memory* (LSTM) cells. This approach achieved a good classification accuracy on various datasets and is therefore also evaluated in this work. The third group of classification algorithms are event-based methods. Information is represented as a temporal pulse sequence and propagated by a spiking neural network (SNN). Consequently, no preprocessing of the raw event data is necessary. Due to the non-differentiability of spikes, the development of learning algorithms is a major challenge. In this work, the network architecture and the learning algorithm from in [20] are evaluated on the new datasets.

This paper adds upon the reviewed literature by covering a complete, modular processing pipeline from raw event data to object classification. The individual modules implement the most recent and promising algorithms in the literature suitable for automatic visual inspection tasks. A major challenge in the classification of event streams is to find the optimal time window at which most object characteristics can be seen. Often, classification is performed multiple times over a large time interval and the results are then fused, such as in [18]. Other approaches use peak detection units [21] to pass information only at relevant times. In this paper, we present a novel method that determines an optimal point of time based on contrast. It takes the density of events with different polarity into account. By reducing the stream to the relevant time interval, the computational effort can be decreased and the correct classification rate significantly increased in challenging scenarios.

### 2.2. Event-Based Datasets

Generally, in recent years, data-driven methods have become increasingly popular for classification tasks and keep superseding conventional image processing methods. Besides high performance in terms of classification accuracy, such methods do not rely on the formulation of explicit rules but rather learn classification rules based on examples that they are presented during a training phase. This trend does not only hold true for frame-based image processing, but also in the context of event-based vision. However, it results in a strong urge for domain-specific datasets, that are used for training and testing of the methods. At the beginning of event-based research, event-based cameras were used to re-record projections of existing, conventionally recorded scenes to create datasets, such as in [22]. This approach was simplified using emulators that generate an event stream from a sequence of conventional frames. One of the most recent approaches in this regard is the method of Delbruck et al. [23]. In addition, real event-based datasets have also emerged, although these almost exclusively contain scenarios in the field of autonomous driving, such as in [18,24]. Previous publications have focused primarily on robotics, autonomous driving, and surveillance applications. A dataset for automatic visual inspection is not yet publicly available but indispensable in order to evaluate applicability of this new sensor type in this field of application. This work aims to fill this gap and initiate more in-depth research in a new application field.

## 3. Event-Based Vision Pipeline

**Figure 2:**
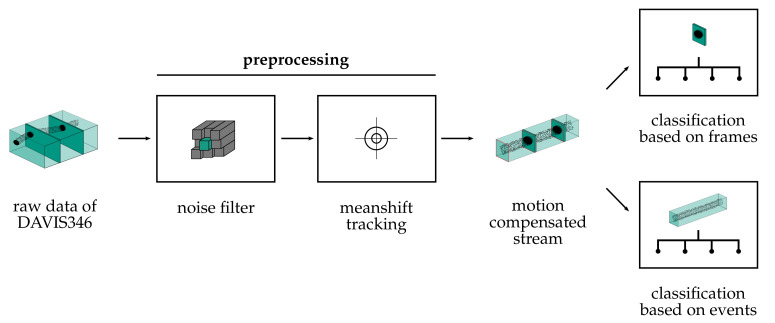
Overview on the modular pipeline used to classify objects based on intensity images and events. The DAVIS346 camera records conventional frames at a constant frame rate and an asynchronous event stream of the moving object in the camera’s FoV. The event stream is denoised by a spatio-temporal filter and a meanshift tracking algorithm determines the object’s centroid based on events only. All information about frames and events is compressed to an ROI formed around the object’s center which leads to a compensation of lateral motion. Based on this, different classification methods are applied and compared.

We propose a modular processing pipeline for object classification using DAVIS cameras. A general overview of the processing steps is provided in Figure 2. The procedure is divided into two stages. The first stage addresses the tasks of preprocessing and object tracking. First, noisy events are filtered out of the stream. Then, a tracking algorithm clusters events of an object and tracks the object center in the image plane. This results in motion-compensated event streams, conventional frames of the DAVIS camera within a region of interest (ROI), and information about the object’s position and velocity. In the second stage, the classification of the detected objects is performed. For this purpose, we consider classification based on frames with conventional methods and also classification utilizing the motion compensated event streams. For the latter, four different approaches are considered. One method is the reconstruction of an intensity image and application of a classical image classifier. Furthermore, HATS [18] and MatrixLSTM [19], two recently proposed methods, are implemented to transform events into a suitable feature space. Based on this, a convolutional neural network with ResNet [25] architecture is used as a classifier. Finally, a SNN is considered that can process the event stream directly, thus completing the neuromorphic approach of event-based cameras. In the following, all components of the pipeline are explained, starting with the format of raw event data.

### 3.1. Event Representation

The pixels of an event camera detect changes in the logarithmic intensity signal independently and emit an asynchronous event stream. According to the address-event representation [4], events are described as tuples

(1) $$e_{k}=\left(x_{k},y_{k},p_{k},t_{k}\right)\;,$$

where $x_{k}$ defines the horizontal and $y_{k}$ the vertical position in the image plane, $p_{k}\in \left\{-1,1\right\}$ the polarity, and $t_{k}$ the timestamp of an event, commonly expressed in microseconds.

Assuming the brightness constancy assumption and a constant illumination, it can be derived that events occur at moving edges [1]. If the edge is aligned parallel to the direction of movement, no events are generated, but if it is aligned perpendicular to it, a maximum number of events are generated. Consequently, only partial areas of a pattern can be perceived that do not have a parallel orientation.

### 3.2. Noise Filtering and Tracking

Pixels with strong activity, so-called hot pixels, are detected and eliminated from the stream during a learning phase while viewing a rigid scene. This learning process only needs to be performed once during the initial camera calibration. To reduce background noise, the well-established spatio-temporal correlation filter from in [9] is used. Partitioning events in packets of 500 μs has been empirically determined to yield a good trade-off between processing quality and speed. Based on the filtered event packets, the tracking algorithm of Barranco et al. [11] is used to determine and track the center of the object. The method is based on a meanshift approach with multivariate Gaussian kernel according to

(2) $$K_{G}\left(x\right)=\frac{1}{\sigma \sqrt{2\pi }}\exp^{-\frac{1}{2}\left(x^{T}Qx\right)}.$$

The variable x is composed of the event position (x,y) and a weighted temporal difference between the timestamp *t* of the event and the end time of the time interval under consideration and Q is a suitable weighting matrix. The clustering result provides the cluster centroid in the image plane and the allocation of the events to the objects. In general, this also allows for multi-object tracking, but in this work we restrict ourselves to tracking one object. The detected object center point is then estimated using a Kalman filter, which includes the position as well as the velocity of the object as part of the state variable. A square ROI of constant size is formed around the object center, which encloses the entire object. To compress information, all events associated with the object are represented relative to this ROI, and the entire stream is limited to the time range in which the object is fully visible. In addition, all conventional frames of the DAVIS camera are also extracted within the ROI. These data form the basis for the subsequent procedures.

### 3.3. Contrast-Based Windowing (CBW)

For both datasets presented in Section 4, the objects are only completely visible in the camera’s field of view for a certain time interval. During this time, the objects can rotate strongly and thus the texture is not constant for all samples at all times in the stream. This challenge distinguishes our dataset strongly from already published ones, as, due to the fields of application yet considered in the literature, rotation of objects rarely occurs and lateral motion is typically much slower. Previously developed classifiers for event streams hardly include this additional degree of freedom.

**Figure 3:**
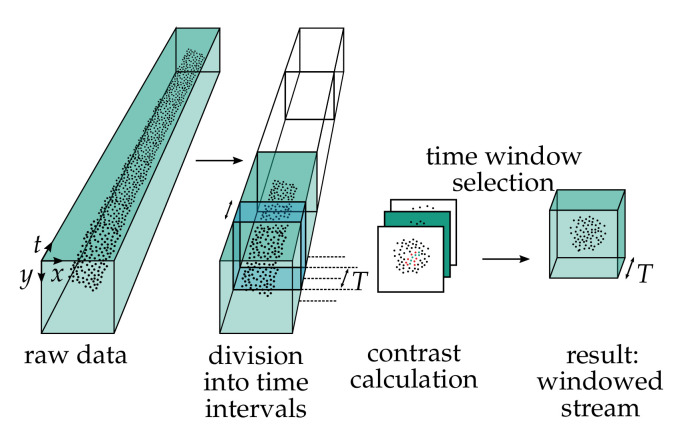
Windowing method to reduce the event stream to the time interval with the highest contrast. The whole event stream is divided into equal time intervals. Within each interval a sliding time window is used to select events for contrast calculation. The contrast is defined as the sum of events of different polarity in a spatial neighborhood. Finally, the time window with the highest contrast in each interval is selected for further processing.

In the context of this study, we observe that a classifier provides higher classification accuracy when the stream is limited to a short time interval. For this reason, this paper presents a method to determine an optimal time interval for classifying an event stream as shown in Figure 3. The basic idea is to detect the time point with maximum contrast. The event stream is divided into *N* time intervals similar to the work in [18,19]. The time of maximum contrast is determined separately for each time interval. For this purpose, a sliding time window of length *T* is applied to the time interval. Within this window, a neighborhood defined by

(3) $$N_{\left(z\right)}\left(e_{i}\right)=\left\{e_{j}:x_{j}=x_{i}+z,p_{i}\neq p_{j}\right\}$$

of an event $e_{i}$ is considered, where $z={\left[-\rho ,\rho \right]}^{2}$ defines the size of this neighborhood and $x_{i}$ describes the position of $e_{i}$. Using this neighborhood, a contrast value *c* is determined according to

(4) $$c=\sum_{i=1}^{N}c_{i}\;c_{i}=\left\{\begin{array}{cc} 1 & \exists \;e_{j}\in N_{\left(z\right)}\left(e_{i}\right)\\ 0 & \mathrm{else} \end{array}\right.\;.$$

Here, *c* is the sum of all events with at least one event of opposite polarity in the neighborhood of ρ. Over all time windows considered, the window that has the largest contrast *c* is chosen. The algorithm splits the original event stream and returns only events within the optimal time window for each time interval. This data is now used as the input signal for the classification algorithms described below.

### 3.4. Classification Framework

To obtain a broad analysis of the suitability of event-based cameras for automatic visual inspection, different classification approaches are tested. We implement a unified framework that allows easy adaptation of the processing pipeline, for example, switching classification methods comparable to the work in [19]. Besides conventional intensity frames that serve as a reference, we consider four approaches. These are described in the following in more detail. A basic distinction is made between frame-based and event-based methods.

#### 3.4.1. Frame-Based Methods

The group of frame-based methods includes all methods in which a frame represents the data basis for the subsequent classification. This frame can be interpreted as a gray scale image of the scene. First, this category includes the conventional images of the DAVIS camera. This approach only serves as a baseline and comparison to a conventional camera with 25 frames per second (fps). Second, the method of Scheerlinck et al. [15] as formalized in Equations (5) and (6) is used to reconstruct an intensity image from the event stream. Although this approach originally applies a complementary filter to fuse event and frame information, it also allows a reconstruction based on events only. To estimate the brightness $\hat{L}$ at a location x, Scheerlinck’s approach reduces to a differential equation as given by

(5) $$\frac{\partial }{\partial t}\hat{L}\left(x,t\right)=E\left(x,t\right)-\alpha \hat{L}\left(x,t\right)$$

where

(6) $$E\left(x,t\right)=\sum_{i=1}^{\infty }p_{i}\sigma \delta \left(t-t_{i}\right)$$

is an accumulated event image and α defines the cut-off frequency. The tuning parameters σ and α are used to ensure the visibility of the object’s texture during a chosen interval length. The initial gray value gradient is chosen to be 128, which corresponds to half of the 8-bit coded value range. As we are focusing on event-based approaches, no information from conventional frames is considered in our work. Reconstructed frames are normalized and then classified with a ResNet-18 [25].

#### 3.4.2. HATS

In addition to frame-based methods, this work applies two approaches that do not reconstruct a grayscale image, but instead transform the events into a feature map. The HATS algorithm [18] divides the image plane into *K* cells of equal size. Incoming events are uniquely assigned to a cell based on their position and polarity. Within the cell, a *Local Memory Time Surface* as defined by

(7) $$T_{e_{i}}\left(z,q\right)=\left\{\begin{array}{cc} \sum_{e_{j}\in N_{\left(z,q\right)}\left(e_{i}\right)}\exp^{-\frac{t_{i}-t_{j}}{\tau }} & \mathrm{if}\;p_{i}=q\\ 0 & \mathrm{else} \end{array}\right.$$

is calculated for each event, where $N_{\left(z,q\right)}$ denotes the neighborhood of an event $z={\left[-\rho ,\rho \right]}^{2}$ over a time interval of length *T* and τ is a time weight. Then, all *Local Memory Time Surfaces* are accumulated and normalized by the number of events within the cell. Due to the superposition of time surfaces, the influence of noisy events can be reduced and the normalization provides additional independence from the number of events and thus the contrast conditions of the scene. Finally, the histograms of the individual cells are combined to form a feature map. In the original publication, a linear support vector machine (SVM) is used for classification. However, in this work it is shown that better results can be obtained using deep neural networks. A ResNet-18 is used for the classification of the time surfaces, which also ensures comparability to the other considered methods.

#### 3.4.3. MatrixLSTM

In addition to HATS, another algorithm that has a similar basic principle is evaluated. Instead of clustering the image plane into cells, MatrixLSTM [19] represents each pixel with its own LSTM cell. For an incoming event in the time interval τ, a feature vector $f_{i}^{x,y}$ is computed based on polarity, other events in a local neighborhood, and several temporal information. All computed features of a pixel are processed with an LSTM cell that combines event dynamics into an output vector $s_{t}^{\left(x,y\right)}$. After the last event of the pixel has been processed in the time interval δt, the last outputs of the LSTM cell $s_{T}^{\left(x,y\right)}$ map the complete event sequence dynamics at the pixel under consideration. The final feature map $S_{\varepsilon }$ is subsequently composed of all outputs of cells $s_{T}^{\left(x,y\right)}$ at their original pixel position. In their paper, Cannici et al. [19] present different configurations that define the structure of the classifier and the selection of features. In the context of our work, we evaluate these different configurations and conclude that the configuration referred to as *ResNet18-Ev2Vid* outperforms the other ones. Thus, we focus on this configuration, by which MatrixLSTM is configured to provide three channels at the output and processes the entire stream as one time interval. The normalized timestamp difference between successive events at a pixel is chosen as the temporal feature. In addition, the polarity of the events is added as a feature.

#### 3.4.4. Multi-Level SNN

Last, a direct end-to-end classification procedure that retains the event character from generation to classification is evaluated. The approach of Liu et al. [20] is used, which is based on a multi-level SNN. Incoming events are convolved in the input layer with Gabor filters that detect patterns of different orientation and scaling. The pulses are integrated with *Leaky Integrate-and-Fire* (LIF) neurons and passed on as a pulse to the next layer as soon as the activity of a cell’s neurons exceeds a threshold. The subsequent layer receives the pulses, and the assignment of cells to neurons is unique. Subsequently, a layer of fully linked synapses enables the learning of different classes based on the detected features.

As a learning algorithm, the authors present a new method called *Segmented Probability-Maximization* (SPA). The algorithm is based on a *Noisy Softplus* feature that is biologically inspired and tailored to the LIF neurons used. The learning algorithm adjusts the weights based on the output neuron potentials and the impulse behavior of the middle layer. The procedure can be summarized in two steps. In the first step, the time point with maximum activity within a specified search radius is determined for each output neuron. Subsequently, the individual weights are adjusted by taking the firing rate of each output neuron into account. The goal is to adjust the weights in a way that the output neuron $n_{j}$ shows the highest activity compared to the other output neurons when viewing an object of class *j*.

As the original source code is not available at the time of this research, the algorithm itself is implemented based on *BindsNet* [26] and integrated into the modular pipeline. Compared to the original release, a few changes are made. The size of the Gabor filter bank is reduced such as there are only four filters of different orientations. A separate consideration of the scaling is not necessary in our use case, because the objects are almost the same size in the image. Reducing the number of filters decreases the computational effort and thus significantly reduces the time for a training run. The filter bank is implemented using a combination of two layers in *BindsNet*. The number of output neurons is adjusted to the number of classes in the dataset. The learning algorithm now adjusts the connection weights between the middle and the output layer.

### 3.5. Summary of the Resulting Pipeline

**Figure 4:**
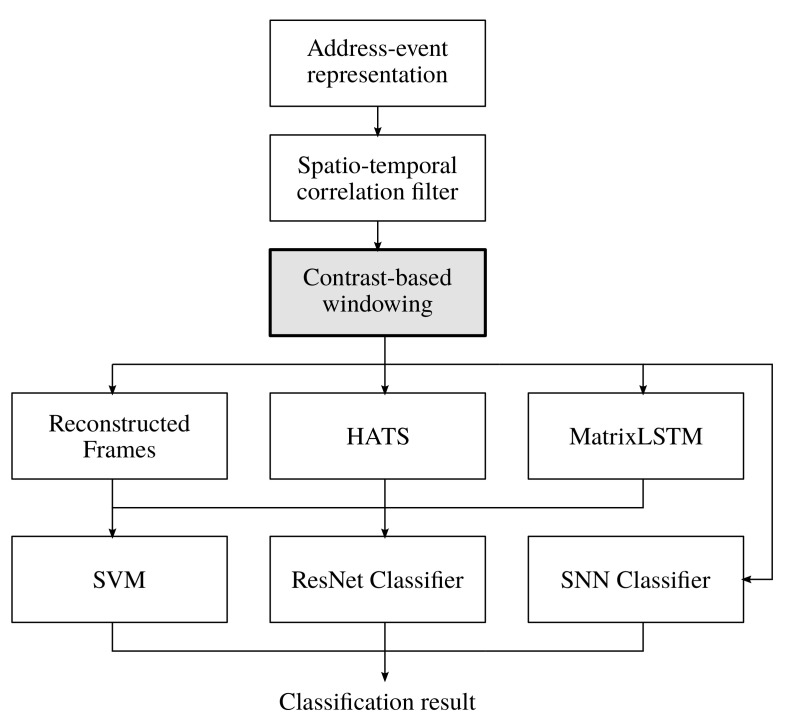
Visual summary of the proposed pipeline including the new CBW approach.

A visual summary of the proposed pipeline is provided in Figure 4. Regarding the input format, the address-event representation [4] is chosen. Noise filtering is implemented using the spatio-temporal correlation filter from in [9]. For object tracking, we use the algorithm from in [11] in order to determine the center of an object together with a Kalman filter. Subsequently, the proposed CBW approach is applied. With respect to classification, two paths exist. For the first path, we integrate the image reconstruction approach from in [15], HATS [18] and MatrixLSTM [19] to calculate feature maps which are then used in conjunction with a SVM or ResNet classifier. For the second path, data are directly classified by a SNN [20].

## 4. Datasets for Visual Inspection

As described in Section 2.2, existing datasets mainly address the fields of autonomous driving, robotics, and surveillance. In order to test an application of the pipeline described above in the application field of automatic visual inspection, novel datasets are generated in this work. Some preliminary considerations are made when selecting suitable scenes for the generation of event-based datasets. In general, scenes of automatic visual inspection are characterized by high dynamics of objects in a controlled environment. The illumination and the distance between camera and object can be assumed to be constant. Especially suitable scenarios that are often found in automatic visual inspection include the inspection of objects during free fall in front of a static background or on a conveyor belt with constant speed. An advantage of the event-based technology is directly shown by the fact that only dynamic and high-contrast image areas are perceived. This greatly simplifies the detection of the objects and computations only need to be made at relevant time intervals. In order to cover a wide range of applications, two different datasets are generated.

**Figure 5:**
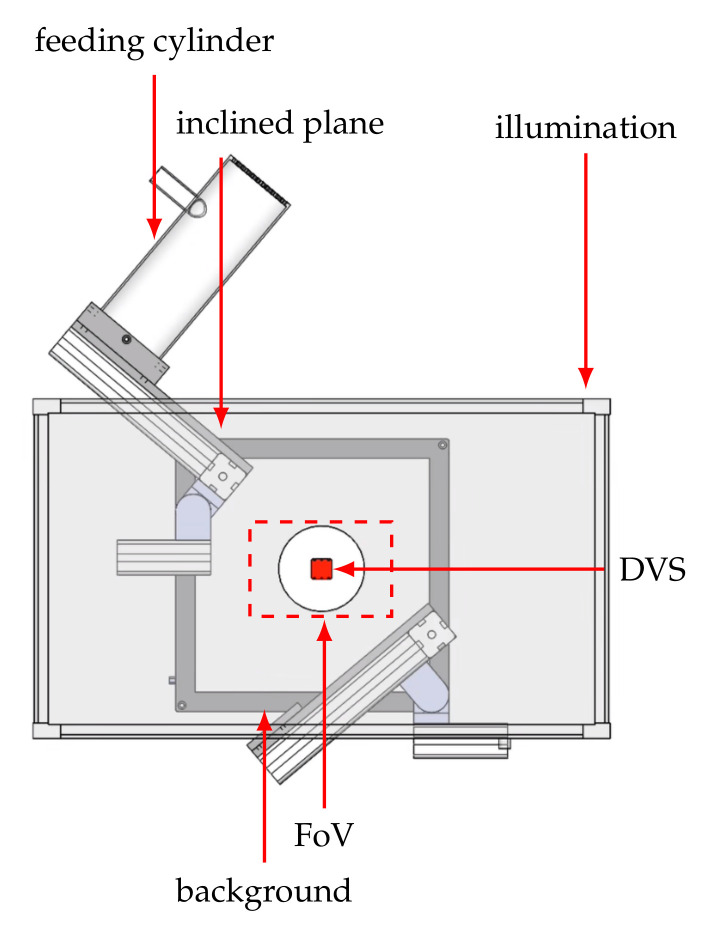
Experimental setup to generate the event-based ball dataset. The illumination panel is shown transparent for a better overview. Starting from the upper cylinder, the balls roll over an inclined plane and cross the camera’s FoV diagonally. As the balls are in free fall they rotate and the camera is able to perceive the object’s pattern in motion. In order to record a large amount of data the cycle is automated by pneumatic conveyance, returning the ball back to the starting point.

**Figure 6:**
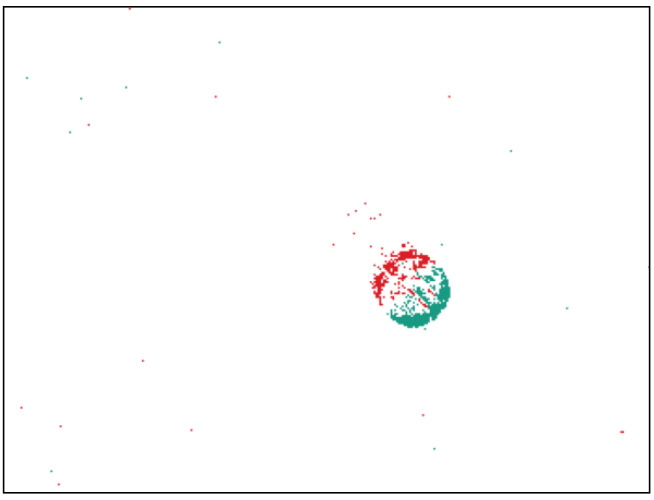
Example of an unprocessed recording of a ball with two stripes during free fall (animated in the digital version of this manuscript).

The first dataset contains recordings of wooden balls with a diameter of 10 mm that differ in their texture by means of a varying number of stripes. We use four balls that are marked by hand with stripes that extend around the complete circumference. The number of stripes varies between none and three, resulting in four different classes to be distinguished. These four test objects are fed repeatedly to the system for data recording. Figure 5 shows the experimental setup which is used to record the balls in free fall. The setup is inspired by the authors of [27]. Starting from the upper cylinder, the balls pass through a small opening onto an inclined plane. There they are accelerated and pass diagonally through the camera’s field of view, see Figure 6. A funnel catches the ball at the bottom and a pneumatic conveying system transports it back into the upper cylinder. The scene is diffusely illuminated using a LED flat dome and the black background ensures that the object is shown with a high contrast. The balls are on average 58 ms completely visible in the image and have an approximate velocity of 1.3 ${\mathrm{ms}}^{-1}$ at the time of disappearance. The particular challenge of the dataset is the strong rotation of the objects, which means that the stripes can only be seen completely at short time intervals.

As a result of the rotation of the balls, the texture may not be fully visible. In order to ensure a distinct database, samples of balls with one, two, or three stripes that do not contain the relevant texture are removed manually. Due to the time-consuming manual process, we restrict ourselves to selecting 2000 samples per class. For the balls without a stripe, we randomly select 2000 samples. The final dataset hence includes 8000 samples.

**Figure 7:**
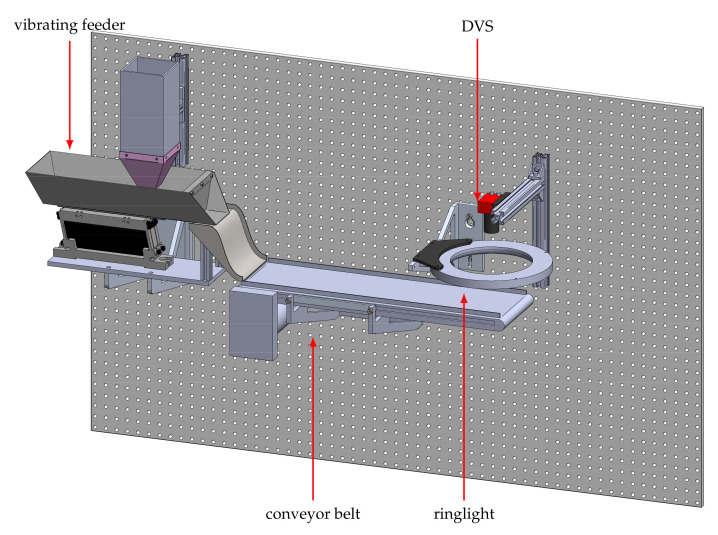
Experimental setup to generate the event-based bean dataset. Two different types of beans are considered: white beans without a pattern and *Borlotti* beans that are spotted with red dots and random texture. Being spread out by the shaker, the beans move towards the conveyor belt successively. Once a bean slid down the ramp the belt conveys it through the camera’s FoV at a speed of approximately $1.1\;{\mathrm{ms}}^{-1}$.

**Figure 8:**
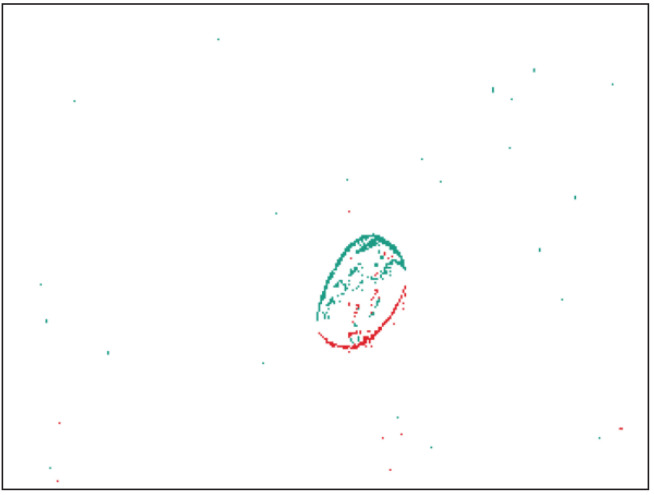
Example of an unprocessed recording of a Borlotti bean on a conveyor belt (animated in the digital version of this manuscript).

The second dataset contains recordings of two bean varieties that differ in texture. The aim is to distinguish white beans from Borlotti beans, which have a cream base color with a random pattern of red dots and stripes. We use approximately 9500 beans of each class for recording data, with each bean passing through the system only once. The second experimental setup is shown in Figure 7. It is adapted from an experimental platform for sensor-based sorting and is described in detail in [28]. The beans are fed into the system by a vibrating feeder onto a conveyor belt via a connecting feeding chute. As soon as a bean enters the belt, it is transported through the field of view of the event-based camera at a speed of $1.1\;{\mathrm{ms}}^{-1}$, see Figure 8. One major challenge of the setup is the moving background. Due to the high dynamic range of event-based cameras, even low contrasts of the black belt can lead to background noise. The scene is illuminated with a diffuse LED ring light. The final dataset includes 18,804 samples, 9353 of Borlotti and 9451 of white beans.

Both scenes are recorded with the event-based camera DAVIS346 by iniVation. Properties of this model can be found in [1]. The parameters of the photoreceptor amplification (PrBp) and source follower (PrSfPb) are tuned manually beforehand such that the object’s texture and outline is clearly visible. Besides the event information, conventional frames are recorded at a rate of 25fps.

**Figure 9:**
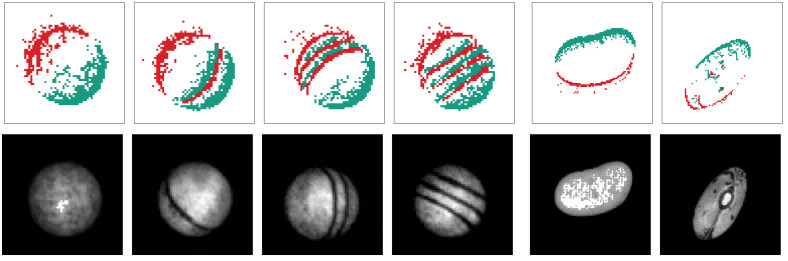
Samples of the preprocessed ball (**left side**) and bean dataset (**right side**). All data have been recorded with a DAVIS346 and preprocessed by the event-based pipeline presented in this paper. After an initial noise filtering, a tracking algorithm based on events only tracks the object’s center. All events and frames of a detected object are compressed to an ROI of constant size around the center. The upper row shows the resulting event stream where positive events are marked in green and negative in red (animated in the digital version of this manuscript). In the lower row sections of conventional gray scale images of the DAVIS camera are displayed that have been extracted by the pipeline.

All raw data are then preprocessed using the event-based pipeline presented in Section 3. In order to filter out background activity noise, we require an event to have at least 2 supporting events in its direct neighborhood during a time horizon of 2000 μs for the ball and 500 μs for the bean dataset. The tracker detects an object as soon as a minimum of 50 events form a valid cluster in case of the ball dataset and 250 events that are validated at least five times in case of the bean dataset. The events and conventional frames of all tracked balls are then reduced to an ROI of size 60×60 pixels and the beans’ data to a window of size 90×90 pixels. A sample of preprocessed events and frames for each class is shown in Figure 9. Beside the frames and events, information about the object’s velocity and actual position is recorded as well. The datasets with all relevant parameter configurations are made publicly available alongside this publication.

## 5. Results

In the following, the performance of the presented data processing methods and the impact of the proposed CBW approach are comparatively evaluated on the basis of the two datasets from Section 4. For our analysis, we split the datasets into a training and a test set with a ratio of 80/20%. As a figure of merit, we use *accuracy* which is calculated on the basis of the resulting confusion matrices as discussed below. It is defined as the ratio of number of samples along the main diagonal of the matrix to the overall number of samples.

**Figure 10:**
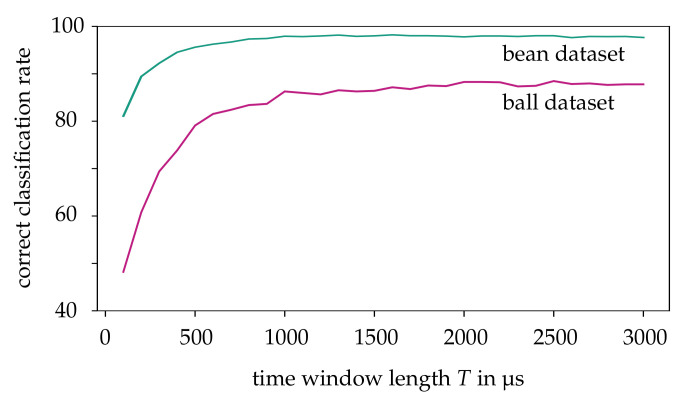
Correct classification rate for both datasets using the contrast-based time windowing with different time window lengths. In this case, the HATS approach with a SVM is used for classification.

We start by elaborating the impact of our CBW approach in more detail. For this purpose, the HATS algorithm with a SVM as proposed in [18] is used as a proven classification method. Due to the low local resolution of the event data, the size of the cells K=3 and the neighborhood ρ=1 is chosen as small as possible. The CBW approach requires two design parameters to be specified, namely, the number of time intervals *N* and the time window length *T*. Empirically, *N* has been shown to have little effect on the correct classification rate and is therefore set to N=1. The resulting classification quality is shown for increasing time window lengths *T* in Figure 10. The purpose of this consideration is to find an optimal *T* that satisfies the trade-off between a high correct classification rate and low observation time. For the following analysis, T=2500 μs for the ball dataset and T=1500 μs for the bean dataset is identified as a reasonable choice. We now compare the classification accuracy of CBW with a randomly selected time window of same length within the stream as shown in Table 1. For the ball dataset, the CBW approach achieves a correct classification rate of 89%, which is a significant improvement over a random time window with a rate of 68%. Thus, selection based on contrast represents a suitable method to reduce streams with highly rotating objects to the essential time interval. A small improvement of 0.3 p.p. on average can also be achieved for the bean dataset. Hence, we conclude that the CBW approach achieves a general benefit.

Following that, the performance of each classification method extended by the CBW approach is determined. The obtained results are summarized in Table 2. The first three methods—intensity frames, reconstructed frames, and HATS—were implemented by ourselves during this work and use a ResNet-18 classifier. To avoid under- and overfitting, an early stopping procedure is used that takes 10% of the training data as the validation dataset. The training is terminated as soon as the classification performance does not increase over 5 epochs or the maximum number of 50 epochs is reached. An Adam optimizer with a learning rate of $1\times 10^{-4}$ and a cross entropy error function is used to optimize the network. In case of MatrixLSTM, the original implementation as provided by Cannici et al. [19] is applied to the full event streams. The CBW method is not used because MatrixLSTM already takes different time intervals and a learning of the time horizon with LSTM cells into account. When using SNN as a classifier, the stream is first reduced using the CBW method and then transformed into a three-dimensional matrix of size x×y×t. The time constant $\tau_{m}$ of the LIF neurons and the search range $t_{r}$ is fixed to the length of the time window *T*. As described in [20], the neuron threshold of the output layer is chosen to be 2 in the training and 1 in the testing phase.

**Figure 11:**
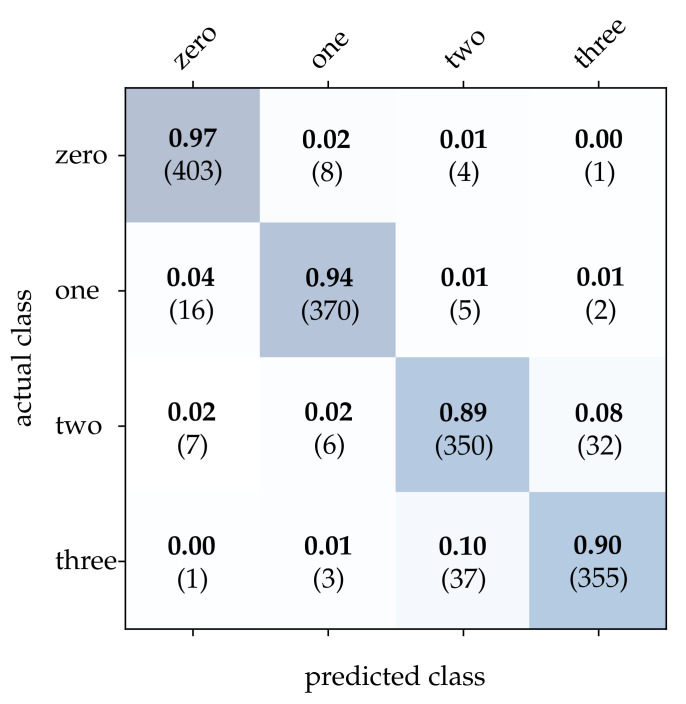
Classification results using image reconstruction and the wooden balls dataset. The bold values denote the relative frequency, the number in brackets the absolute number of samples.

**Figure 12:**
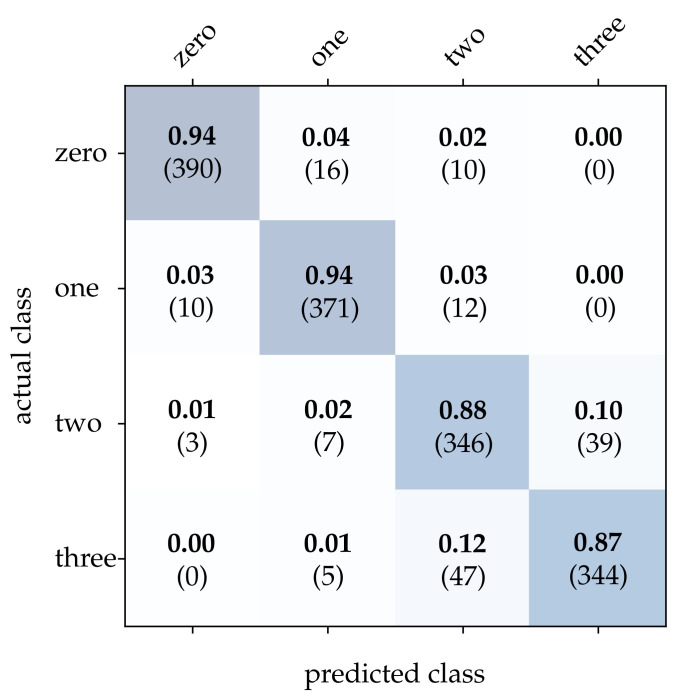
Classification results using HATS and the wooden balls dataset. The bold values denote the relative frequency, the number in brackets the absolute number of samples.

**Figure 13:**
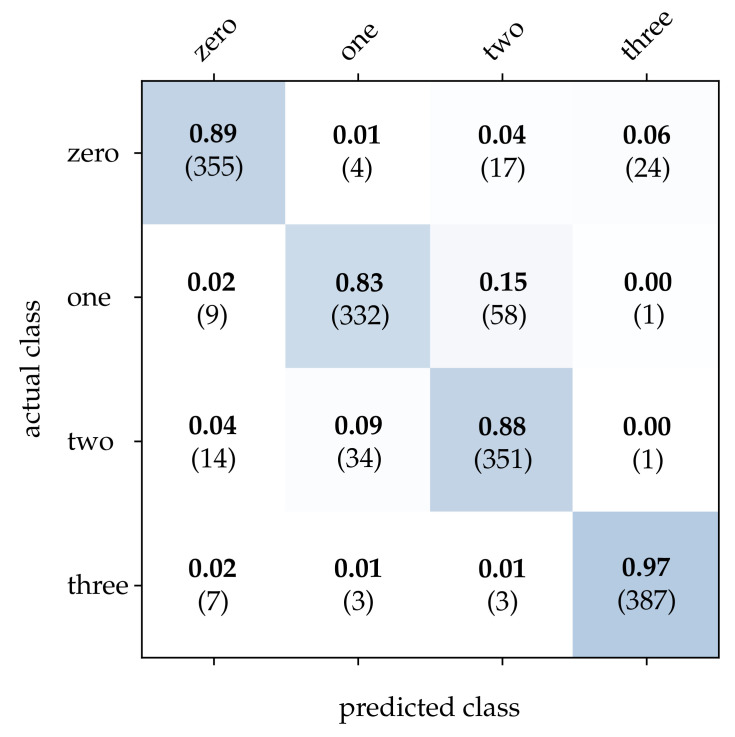
Classification results using MatrixLSTM and the wooden balls dataset. The bold values denote the relative frequency, the number in brackets the absolute number of samples.

**Figure 14:**
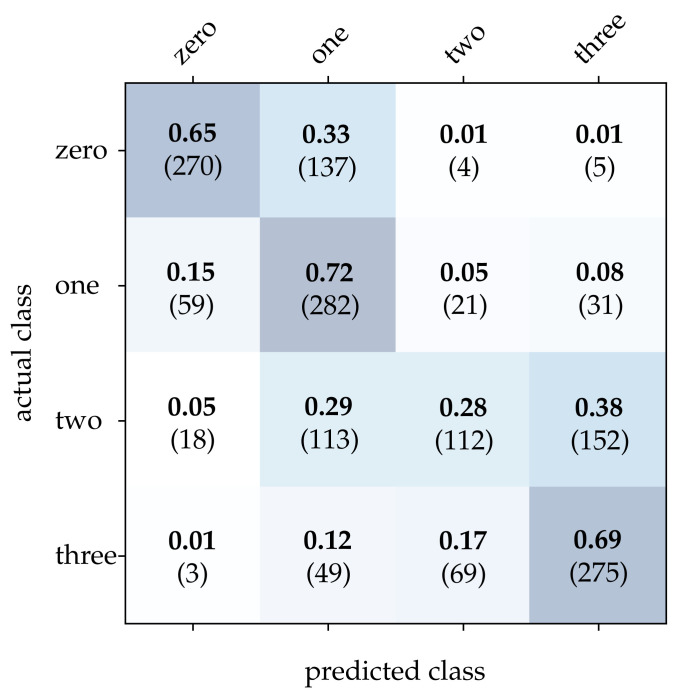
Classification results using the SNN and the wooden balls dataset. The bold values denote the relative frequency, the number in brackets the absolute number of samples.

First, we take a closer look at the wooden ball dataset. As can be seen from Table 2, conventional frames achieve a mediocre correct classification rate compared to the other approaches, which can be attributed to a frame rate that is too low for the task. It can be expected that a higher sampling rate of the scene will improve the result significantly. Detailed results for image reconstruction [15] and HATS [18] are provided in Figure 11 and Figure 12. As can be seen, using these methods results in particularly high accuracy for the detection of balls with no or only one stripe. However, MatrixLSTM [19] achieves the highest accuracy for the detection of balls with four stripes, see Figure 13. The result of the SNN [20] is clearly overshadowed by the other methods, see Figure 14. This is due to a low network depth and the performance of the SPA learning algorithm. Due to the high event activity at the contour of the balls, the SNN obtains a large amount of irrelevant data for the classification and fails to extract the essential information.

Both the image reconstruction and the HATS method use the proposed approach for selecting the time interval with maximum contrast. These methods also achieve the highest accuracy for this dataset. This further demonstrates that CBW is a suitable method to increase the quality of the classifier significantly. A determinant difference between the image reconstruction and the HATS method is the local averaging of the events. As the resolution of 60×60 pixels is low for the detection of the stripes, the local averaging of the HATS algorithm additionally decreases the visibility of the stripes. However, this can also be observed with MatrixLSTM, which leads to the conclusion that a higher local resolution would achieve a better result over the two methods.

**Figure 15:**
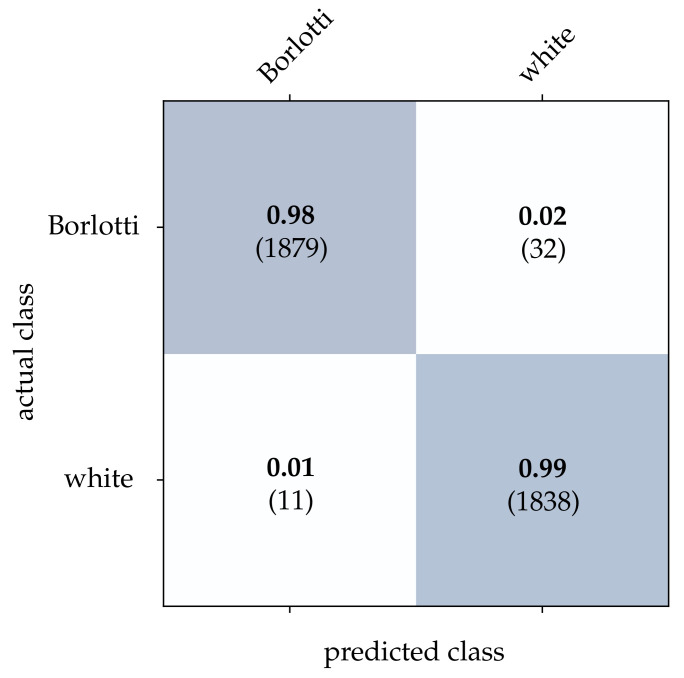
Classification results using image reconstruction and the beans dataset. The bold values denote the relative frequency, the number in brackets the absolute number of samples.

**Figure 16:**
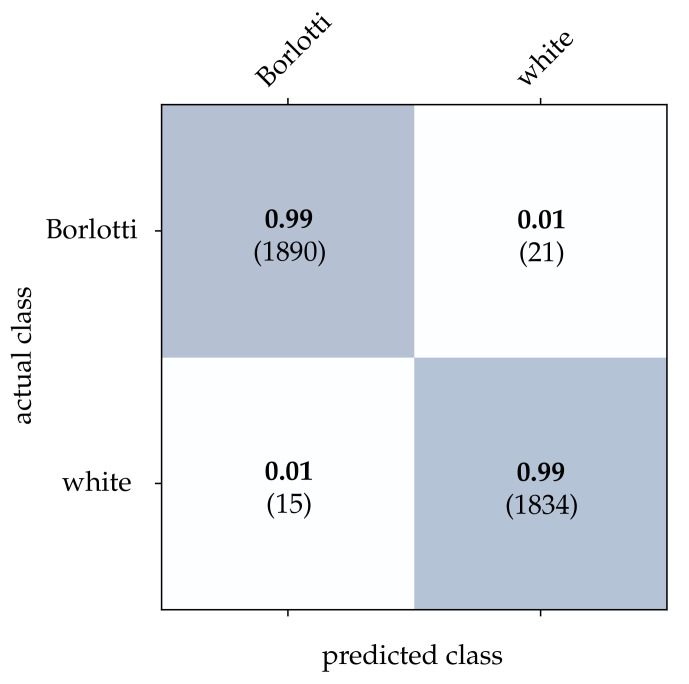
Classification results using HATS and the beans dataset. The bold values denote the relative frequency, the number in brackets the absolute number of samples.

**Figure 17:**
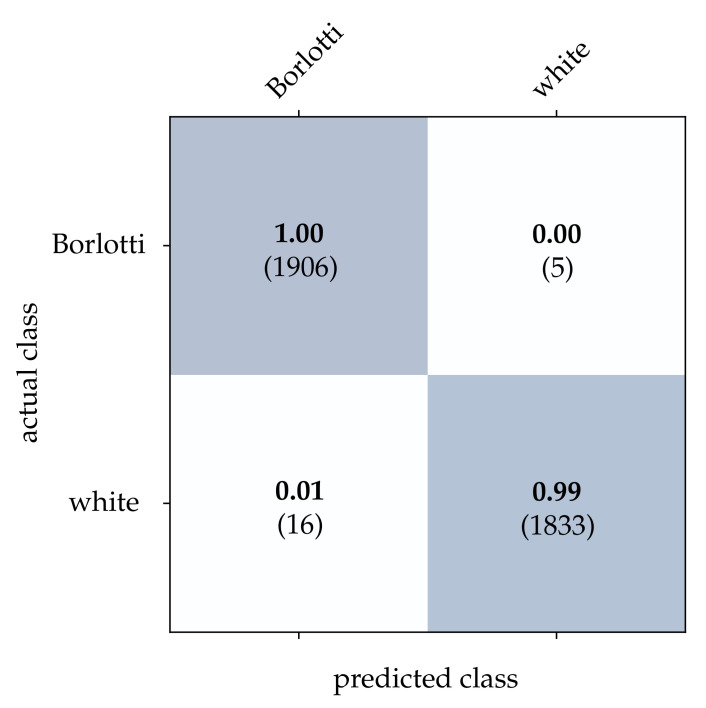
Classification results using MatrixLSTM and the beans dataset. The bold values denote the relative frequency, the number in brackets the absolute number of samples.

**Figure 18:**
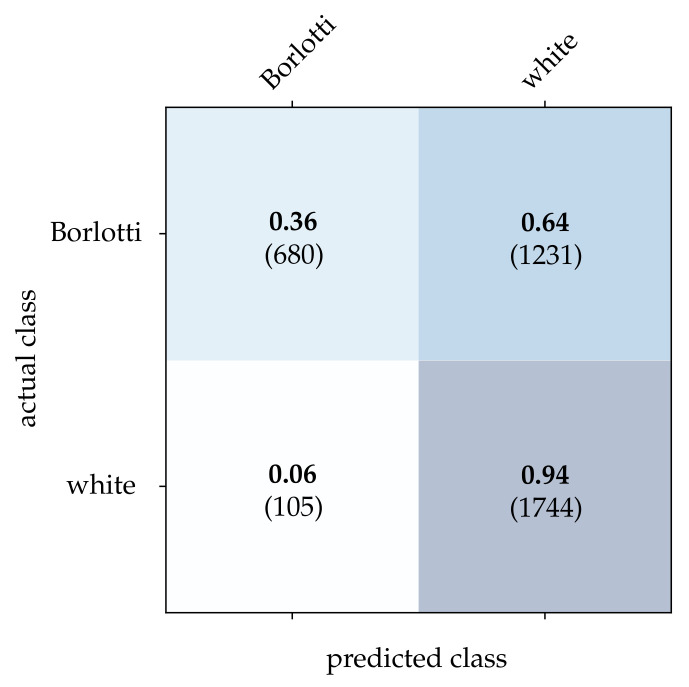
Classification results using the SNN and the beans dataset. The bold values denote the relative frequency, the number in brackets the absolute number of samples.

For the bean dataset, it can be seen from Table 2 that a correct classification based on the conventional images is clearly possible. However, HATS [18] and MatrixLSTM [19], which both transfer the event stream into a feature space, come very close to this result. Due to the higher resolution of 90×90 pixels, the advantage of methods that accumulate events in a local neighborhood becomes apparent at this point. The reconstruction method [15] only considers the event rate for each pixel independently, and thus it is more prone to noise which leads to a slightly lower correct classification rate. From Figure 15, Figure 16 and Figure 17, it can be seen that all three methods detect both kinds of beans with comparable accuracy. The SNN [20] also achieves comparatively low correct classification rates for the bean dataset, see Figure 18. Due to the larger ROI, significantly more neurons are required per layer, which further complicates the learning algorithm requirements.

Overall, it can be summarized that conventional frames can be used to achieve the best classification results given sufficient sampling of the scene. These methods are already established and well researched. However, feature-based algorithms for processing the events come very close to the conventional frames and offer the benefit to improve the processing speed significantly while reducing the amount of data. Regarding the current state of research, the results of event-based methods such as SNNs cannot keep up with feature-based ones. However, due to the constantly growing research field, further developments for SNN architectures and learning algorithms are to be expected in the near future.

## 6. Conclusions

In this paper, we presented a modular pipeline including the common processing steps in machine vision for the new DVS principle. For the evaluation of state-of-the-art processing algorithms as well as a newly introduced algorithmic approach, we introduced two novel datasets for the so far unconsidered field of application of automatic visual inspection. The first dataset includes samples of four types of wooden balls and contains 8000 samples. The wooden balls feature one, two, three, or four stripes, resulting in a four-class discrimination problem. The second dataset consists of two types of beans that differ in texture and contains approximately 18,804 samples. Data were acquired in typical visual inspection settings, i.e., on a conveyor belt and during free fall. For this purpose, we considered two different experimental setups, each of which with its own special characteristics. By making these datasets publicly available, we provide a basis for further research on the application of DVS in the context of machine vision.

We introduced a novel algorithmic approach for selecting an ideal time window for object classification within an event stream. This approach provides a solution for dealing with typical challenges in this field of application, for instance object rotations. Based on the introduced datasets, it was shown that this extension can significantly increase the accuracy of proven classification methods. Using HATS [18] in conjunction with a SVM classifier, it was shown that classification accuracy can be increased from 68.37 to 88.69 for the wooden balls dataset and from 97.77 to 98.01 for the beans dataset.

We extended classification methods based on reconstructed frames [15], HATS [18], MatrixLST [19] and a SNN [20] by our novel approach and evaluated their performance on the basis of the new datasets. For the wooden balls dataset, it was shown that reconstructed frames and HATS achieved particularly high classification accuracy of up to 92.37. For the beans dataset, none of the approaches outperformed classification based on conventional intensity frames. However, using MatrixLSTM, a high accuracy of 99.44 was also obtained. Results obtained with the SNN were not able to keep up with the other considered approaches. However, further developments for SNN architectures and learning algorithms could change that.

In the future, we are interested in investigating DVS technology for deriving mechanical properties of test objects. Approaches as considered in visual vibrometry might be exploited in DVS due to the high temporal resolution. However, we consider further advances in sensor technology, especially regarding the spatial resolution, as a necessity for such kinds of tasks. Furthermore, advances in SNN are required in order to design systems according to the neuromorphic concept of the sensors.

## Figures and Tables

**Table 1:** Comparison between the classification accuracy in percent using a random time interval and CBW of the same length using HATS with a SVM as classifier.

Method	Balls	Beans
Random time interval	68.37	97.77
CBW	88.69	98.01

**Table 2:** Classification accuracy in percent of the presented datasets using contrast-based windowing and state-of-the-art classification methods.

Method	Classifier	Balls	Beans
Intensity frames	ResNet-18	81.37	100
Reconstructed frames [15]	ResNet-18	92.37	98.86
HATS [18]	ResNet-18	90.69	99.04
Matrix-LSTM [19]	ResNet18-Ev2Vid	89.06	99.44
SPA [20]	SNN	58.69	64.47

## Data Availability

The data presented in this study will be openly available in Fordatis at http://dx.doi.org/10.24406/fordatis/148 shortly after publication of this article.

## References

[1] Gallego G., Delbruck T., Orchard G.M., Bartolozzi C., Taba B., Censi A., Leutenegger S., Davison A., Conradt J., Daniilidis K. (2020). Event-based Vision: A Survey. IEEE Trans. Pattern Anal. Mach. Intell..

[2] Holešovský O., Škoviera R., Hlaváč V., Vítek R. (2021). Experimental Comparison between Event and Global Shutter Cameras. Sensors.

[3] Beyerer J., Puente León F., Frese C. (2016). Machine Vision: Automated Visual Inspection: Theory, Practice and Applications.

[4] Lichtsteiner P., Posch C., Delbruck T. (2008). A 128x128 120 dB 15 *μ* Latency Asynchronous Temporal Contrast Vision Sensor. IEEE J. Solid-State Circuits.

[5] Brandli C., Berner R., Yang M., Liu S.C., Delbruck T. (2014). A 240 × 180 130 db 3 μs latency global shutter spatiotemporal vision sensor. IEEE J. Solid-State Circuits.

[6] Steffen L., Reichard D., Weinland J., Kaiser J., Roennau A., Dillmann R. (2019). Neuromorphic Stereo Vision: A Survey of Bio-Inspired Sensors and Algorithms. Front. Neurorobotics.

[7] Posch C., Serrano-Gotarredona T., Linares-Barranco B., Delbruck T. (2014). Retinomorphic Event-Based Vision Sensors: Bioinspired Cameras With Spiking Output. Proc. IEEE.

[8] Feng Y., Lv H., Liu H., Zhang Y., Xiao Y., Han C. (2020). Event Density Based Denoising Method for Dynamic Vision Sensor. Appl. Sci..

[9] Delbruck T. Frame-free dynamic digital vision. Proceedings of the International Symposium on Secure-Life Electronics, Advanced Electronics for Quality Life and Society 2008.

[10] Zhenjiang N., Bolopion A., Agnus J., Benosman R., Regnier S. (2012). Asynchronous Event-Based Visual Shape Tracking for Stable Haptic Feedback in Microrobotics. IEEE Trans. Robot..

[11] Barranco F., Fermuller C., Ros E. (2018). Real-time clustering and multi-target tracking using event-based sensors. arXiv.

[12] Zhu A.Z., Atanasov N., Daniilidis K. Event-based feature tracking with probabilistic data association. Proceedings of the 2017 IEEE International Conference on Robotics and Automation (ICRA).

[13] Mitrokhin A., Fermuller C., Parameshwara C., Aloimonos Y. Event-Based Moving Object Detection and Tracking. Proceedings of the 2018 IEEE/RSJ International Conference on Intelligent Robots and Systems (IROS).

[14] Gehrig D., Rebecq H., Gallego G., Scaramuzza D. (2019). EKLT: Asynchronous Photometric Feature Tracking Using Events and Frames. Int. J. Comput. Vis..

[15] Scheerlinck C., Barnes N., Mahony R., Jawahar C., Li H., Mori G., Schindler K. (2019). Continuous-Time Intensity Estimation Using Event Cameras. Computer Vision—ACCV 2018.

[16] Rebecq H., Ranftl R., Koltun V., Scaramuzza D. (2020). High Speed and High Dynamic Range Video with an Event Camera. IEEE Trans. Pattern Anal. Mach. Intell..

[17] Lagorce X., Orchard G., Galluppi F., Shi B.E., Benosman R.B. (2017). HOTS: A Hierarchy of Event-Based Time-Surfaces for Pattern Recognition. IEEE Trans. Pattern Anal. Mach. Intell..

[18] Sironi A., Brambilla M., Bourdis N., Lagorce X., Benosman R. HATS: Histograms of Averaged Time Surfaces for Robust Event-based Object Classification. Proceedings of the IEEE Conference on Computer Vision and Pattern Recognition.

[19] Cannici M., Ciccone M., Romanoni A., Matteucci M. Matrix-LSTM: A Differentiable Recurrent Surface for Asynchronous Event-Based Data. Proceedings of the European Conference on Computer Vision (ECCV).

[20] Liu Q., Ruan H., Xing D., Tang H., Pan G. Effective AER Object Classification Using Segmented Probability-Maximization Learning in Spiking Neural Networks. Proceedings of the AAAI Conference on Artificial Intelligence.

[21] Zhao B., Ding R., Chen S., Linares-Barranco B., Tang H. (2015). Feedforward Categorization on AER Motion Events Using Cortex-Like Features in a Spiking Neural Network. IEEE Trans. Neural Netw. Learn. Syst..

[22] Orchard G., Jayawant A., Cohen G.K., Thakor N. (2015). Converting Static Image Datasets to Spiking Neuromorphic Datasets Using Saccades. Front. Neurosci..

[23] Delbruck T., Hu Y., He Z. (2020). V2E: From video frames to realistic DVS event camera streams. arXiv.

[24] Hu Y., Binas J., Neil D., Liu S.C., Delbruck T. DDD20 End-to-End Event Camera Driving Dataset: Fusing Frames and Events with Deep Learning for Improved Steering Prediction. Proceedings of the 2020 IEEE 23rd International Conference on Intelligent Transportation Systems (ITSC).

[25] He K., Zhang X., Ren S., Sun J. Deep Residual Learning for Image Recognition. Proceedings of the IEEE Conference on Computer Vision and Pattern Recognition.

[26] Hazan H., Saunders D.J., Khan H., Sanghavi D.T., Siegelmann H.T., Kozma R. (2018). BindsNET: A machine learning-oriented spiking neural networks library in Python. Front. Neuroinform..

[27] Maier G., Mürdter N., Gruna R., Längle T., Beyerer J. (2019). Automatic visual inspection based on trajectory data. OCM 2019-Optical Characterization of Materials: Conference Proceedings.

[28] Maier G., Pfaff F., Pieper C., Gruna R., Noack B., Kruggel-Emden H., Längle T., Hanebeck U.D., Wirtz S., Scherer V. (2020). Experimental Evaluation of a Novel Sensor-Based Sorting Approach Featuring Predictive Real-Time Multiobject Tracking. IEEE Trans. Ind. Electron..

